# Modulation of Colorectal Tumor Behavior via lncRNA TP53TG1-Lipidic Nanosystem

**DOI:** 10.3390/pharmaceutics13091507

**Published:** 2021-09-18

**Authors:** Farimah Masoumi, Sofia M. Saraiva, Belén L. Bouzo, Rafael López-López, Manel Esteller, Ángel Díaz-Lagares, María de la Fuente

**Affiliations:** 1 Nano-Oncology and Translational Therapeutics Unit, Health Research Institute of Santiago de Compostela (IDIS), SERGAS, 15706 Santiago de Compostela, Spain; farimah.masoumi@gmail.com (F.M.); sofia.m.s.73@gmail.com (S.M.S.); lopezbouzobelen@gmail.com (B.L.B.); 2 Immunology Department, School of Medicine, Tehran University of Medical Sciences, Tehran 14176-13151, Iran; 3 School of Medicine, Tonekabon Branch, Islamic Azad University, Tonekabon 46841-61167, Iran; 4 Centro de Investigación Biomédica en Red de Cancer (CIBERONC), 28029 Madrid, Spain; rafael.lopez.lopez@sergas.es (R.L.-L.); mesteller@carrerasresearch.org (M.E.); 5 Translational Medical Oncology Group (Oncomet), Health Research Institute of Santiago de Compostela (IDIS), SERGAS, 15706 Santiago de Compostela, Spain; 6 Josep Carreras Leukaemia Research Institute (IJC), 08916 Barcelona, Spain; 7 Physiological Sciences Department, School of Medicine and Health Sciences, University of Barcelona (UB), 08907 Barcelona, Spain; 8 Institucio Catalana de Recerca i Estudis Avançats (ICREA), 08010 Barcelona, Spain; 9 Cancer Epigenomics, Translational Medical Oncology Group (Oncomet), Health Research Institute of Santiago de Compostela (IDIS), University Clinical Hospital of Santiago (CHUS), SERGAS, 15706 Santiago de Compostela, Spain

**Keywords:** long non-coding RNAs, epigenomics, colorectal cancer, nanocarriers, emulsions

## Abstract

Long non-coding RNAs (lncRNAs) are an emerging group of RNAs with a crucial role in cancer pathogenesis. In gastrointestinal cancers, TP53 target 1 (TP53TG1) is an epigenetically regulated lncRNA that represents a promising therapeutic target due to its tumor suppressor properties regulating the p53-mediated DNA damage and the intracellular localization of the oncogenic YBX1 protein. However, to translate this finding into the clinic as a gene therapy, it is important to develop effective carriers able to deliver exogenous lncRNAs to the targeted cancer cells. Here, we propose the use of biocompatible sphingomyelin nanosystems comprising DOTAP (DSNs) to carry and deliver a plasmid vector encoding for TP53TG1 (pc(TP53TG1)-DSNs) to a colorectal cancer cell line (HCT-116). DSNs presented a high association capacity and convenient physicochemical properties. In addition, pc(TP53TG1)-DSNs showed anti-tumor activities in vitro, specifically a decrease in the proliferation rate, a diminished colony-forming capacity, and hampered migration and invasiveness of the treated cancer cells. Consequently, the proposed strategy displays a high potential as a therapeutic approach for colorectal cancer.

## 1. Introduction

Epigenetic mechanisms play an important role in regulating gene expression during many biological processes in normal cells. However, the deregulation of these epigenetic mechanisms in cancer cells has important implications for cancer development and progression, and therapy response [1]. Thus, cancer epigenetics represents a very promising field with clinical utility for precision oncology [2,3]. Most of the studies have been focused on the analysis of the most well-known epigenetic mechanisms, such as DNA methylation and histone modifications. Of note, there is another layer of complexity in cancer epigenetics represented by the expression of different types of non-coding RNAs (ncRNAs), which can be also regulated by other epigenetic mechanisms [4,5]. It has been shown that most of the transcribed RNAs in the cells are ncRNAs, which lack protein-coding potential and only 2% of the transcriptome is translated into functional proteins [6]. Among the several types of ncRNAs, long ncRNAs (lncRNAs) are an emerging group of heterogeneous non-coding transcripts longer than 200 nucleotides that are involved in many biological processes [4]. LncRNAs are important regulators of gene expression networks controlling several functions in the cells including nuclear architecture and transcription, messenger RNA (mRNA) stability, translation, and post-translational modifications. In addition, lncRNAs often contain embedded sequence motifs that can bind proteins to regulate their function through recruitment or sequestration [7,8]. Among the different functions displayed in the cells, lncRNAs can act as tumor suppressors inhibiting cancer development and progression [9,10]. This ability makes lncRNAs potent therapeutic mediators in cancer treatments [11]. Importantly, some lncRNAs have been demonstrated to be associated with the p53 regulatory pathway [9,10], which is one of the major mediators of the tumor suppressor response in the cells [12]. In fact, the expression of the lncRNA TP53TG1 has shown to be induced in a p53-dependent manner [13] displaying a critical role for the correct response of p53 to DNA damage [14]. TP53TG1 has also shown tumor suppressor properties due to its ability to prevent the nuclear localization of the oncogenic YBX1 protein [14], which has been associated with cancer progression [15]. We have also previously demonstrated that TP53TG1 can undergo DNA methylation-associated silencing in gastrointestinal tumors producing aggressive tumors that are resistant to cellular death by DNA-damaging agents [14]. More recently, other studies have shown that lncRNA-TP53TG1 also acts as a tumor suppressor in breast cancer [16] and hepatocellular carcinoma [17] through different mechanisms. Nonetheless, there are also studies that demonstrate TP53TG1 role in promoting the development of other types of cancers such as pancreatic adenocarcinoma [18], glioma (under low glucose levels) and nasopharyngeal carcinoma [19]. Regarding non-small cell lung cancer, Xiao et al. showed that TP53TG1 increases the sensitivity of these cancer cells to DNA damaging agents by modulating miR-18a/PTEN axis [20]. Hence, TP53TG1 therapies could act as an efficient anti-cancer drug.

The main problem when developing a nucleic acid-based therapy is that nucleic acids need to be efficiently protected from degradation and delivered to their target site. In this sense, different synthetic nanocarriers have been developed for gene therapy [21]. Nanocarriers have drawn increasing interest for their special features such as nanoscale size and favorable physicochemical characteristics to deliver drugs in the optimum dosage range to their target site, often resulting in increased therapeutic efficacy of the drug and weakened side effects [22]. By providing protection from nucleases degradation, the nanocarriers keep nucleic acids intact and biologically active while improving their access to the target site and cellular compartment [23]. There are different types of nanocarriers proposed for gene therapy, being cationic liposomes the most widely cited [24,25]. Typically, cationic nanosystems aimed at gene therapy applications incorporate cationic lipids such as 1,2-Dioleoyloxy-3-Trimethylammoniumpropanchloride (DOTAP), N-[1-(2,3-dioleyloxy)propyl]-N,N,N-trimethylammonium chloride (DOTMA 3-b[N-(N0,N0-dimethylaminoethane)-carbamoyl] cholesterol (DC-Chol), stearylamine (ST) or cetyltrimethylammonium bromide (CTAB) to favor the establishment of electrostatic interactions with anionic nucleic acids [26]. Emulsions are promising delivery systems that have a broad number of applications in biomedicine [27]. In a previous study, we observed that DOTAP showed a superior miRNA-complexing capacity than ST, forming stable lipoplexes. In addition, when encapsulating these DOTAP lipoplexes in nanoemulsions, the resultant nanosystems presented significantly higher internalization and transfection efficiencies than nanosystems containing ST [28].

We propose here the development of a nanosystem specifically for the delivery of a plasmid encoding TP53TG1 to mediate a therapeutic effect able to reduce the oncogenic properties of tumor cells. We have recently described the development of nanometric emulsions composed of vitamin E oil stabilized with sphingomyelin (SNs) [28,29,30,31,32], one of the most abundant lipids in the cell membranes [33]. Our SNs present a simple and biocompatible composition with long-term storage and colloidal stability in different biorelevant media. In addition, they have the capacity to carry molecules of hydrophilic and hydrophobic characters [29]. As such, we have studied their feasibility for different health care applications as diagnostic [30,32,34] and disease treatment [28,31].

Herein, we tune their composition by incorporating DOTAP (DSNs) in order to achieve a good association and to deliver a plasmid expressing lncRNA TP53TG1 to colorectal cancer cells, increasing the intracellular levels of this tumor suppressor. Results point out the development of a gene therapy nanomedicine able to interfere with tumor growth and migration/invasion with a promising application for the treatment of colorectal cancer.

## 2. Materials and Methods

### 2.1. Materials

Vitamin E (Vit E, DL-α-Tocopherol) was purchased from Calbiochem (Merck-Millipore, Darmstadt, Germany). 1,2-Dioleoyloxy-3-Trimethylammoniumpropanchloride (Lipoid DOTAP) and Sphingomyelin (Lipoid E SM) were kindly provided by Lipoid GmbH (Ludwigshafen, Germany). C12-NBD Sphingomyelin (NBD-SM) was purchased from Avanti Polar Lipids (AL, USA). Dulbecco’s Modified Eagle Medium (DMEM), phosphate buffer solution (PBS), 4′,6-Diamidino-2-phenylindole dihydrochloride (DAPI), dimethyl sulfoxide (DMSO), and 1-(4,5-Dimethylthiazol-2-yl)-3,5-diphenylformazan (MTT) were purchased from Sigma Aldrich (Madrid, Spain). Fetal bovine serum (FBS), Penicillin/Streptomycin (PenStrep), Trypsine and 1,1′-dioctadecyltetramethyl indotricarbocyanine iodide (DiR) were acquired from Thermo Fisher Scientific (Madrid, Spain). pcDNA 4To-Mito-mCherry was purchased from TriLink (USA) and pcDNA4/TO was provided by Invitrogen. The vector construction for TP53TG1 overexpression was performed as described in detail in a previous work [14]. Briefly, for the TP53TG1 wild-type overexpression, the cDNA sequence of TP53TG1 (NR_015381; GRCh38/hg38) was cloned into the pcDNA4/TO vector (pc(TP53TG1)). TP53TG1 wild-type sequence was point mutated by site-directed mutagenesis and cloned into the pcDNA4/TO vector. This mutation was performed in the second A and fourth C of all the CACC motifs (CACC>CTCG) present in the TP53TG1 wild-type sequence, which are responsible for the high-affinity YBX1 binding (PMID: 22730292, PMID: 23846655).

### 2.2. Preparation and Characterization of Sphingomyelin Nanosystems

Sphingomyelin nanosystems and DOTAP-SNs were prepared by homogenization method using an Ultraturrax VDI 12 apparatus (VWR, Barcelona, Spain). Briefly, Vit E, SM, and in the case of the cationic nanosystems also DOTAP, were dissolved in ethanol and then mixed in an adequate ratio (1:0.1:0.01 to 1:0.1:0.2 Vit E:SM:DOTAP *w/w*, DSNs 1%, 5%, 10% and to 20%). One hundred microliters of this organic phase were transferred into a microtube and 1 mL of miliQ water was rapidly added over it and immediately homogenized for 15 s with the Ultraturrax at 5000 rpm. The isolation of the formulations was done by ultracentrifugation at 35,000 rpm for 1 h at 15 °C (OptimaTM L-90K, Beckman Coulter, Brea, CA, USA). Physicochemical characterization in terms of particle size and homogeneity of the colloidal nanosystems was performed using dynamic light scattering (DLS) and the surface charge was measured by laser Doppler anemometry, using a Zetasizer NanoZS^®^ (Malvern Instruments, Worcestershire, UK). The colloidal stability of pc(mCherry)-DSNs10% was evaluated in DMEM supplemented with 1% of FBS at 37 °C (1:10, *v/v*), under constant horizontal shaking. Particle size was measured up to 4 h using Zetasizer NanoZS^®^.

### 2.3. Association of Nucleic Acids

The association of plasmid vector (pcDNA) to DSNs (pcDSNs) was performed in round-bottom 96-well plates. Briefly, the same volume of nanosystems (100 µL) and nucleic acids (100 µL) were mixed by pipetting several times and then leaving plates for 20 min in an orbital shaker (200 rpm) at room temperature (RT). The concentration of the pcDNA solution was increased to achieve different theoretical loadings (1% with respect to the total mass of DSNs to 10%), A qualitative determination of pcDNA association efficiency to DSNs was performed by gel retardation assay. A solution of pcDNA and pcDSNs (equivalent volume containing 0.2 µg of pcDNA) and SYBR^®^ Gold, was loaded into each well of an agarose gel (1% in Tris-Borate-EDTA buffer). The gel was run for 50 min at 100 mV and finally imaged with a Molecular Imager^®^ Gel Doc™ XR System (Bio-Rad, Hercules, CA, USA). Physicochemical characterization (particle size, polydispersity, zeta potential) was determined as described in Section 2.2 using a Zetasizer NanoZS^®^ (Malvern Instruments, Worcestershire, UK).

### 2.4. Nanosystems Cytotoxicity Profile

Colorectal cancer cell line HCT-116 (ATCC^®^ CCL247™) was cultured in DMEM medium supplemented with 10% (*v/v*) FBS and 1% PenStrep at 37 °C in 5% CO_2_ and passed before reaching confluence. In order to determine the cytotoxicity profile of the nanosystems, HCT-116 cells were seeded in 96-well plates at 1 × 10^4^ cells/well in supplemented DMEM. Twenty-four hours after culture, the cells were incubated with blank nanosystems (SNs and DSNs1% and 10%) as well as pc(mCherry)-DSNs10% (10 µg model plasmid mCherry) at increasing concentrations for 24 h at 37 °C. Afterwards, the cells were washed with PBS and 110 µL of MTT solution (5 mg/mL, Alfa Aesar, Germany) in DMEM was added per well and incubated for 3 h at 37 °C. The formazan crystals were then dissolved in 100 µL of DMSO and the absorbance was measured at 570 nm using an automatized micro plate reader (Multiscan EX, Thermo Scientific). Cell viability (%) was calculated related to control wells, where cells were treated with ultrapure water instead of SNs.

### 2.5. Nanosystems Internalization by Colorectal Cancer Cells

For internalization studies, blank nanosystems SNs and DSNs (1 and 10% DOTAP) were labeled with DiR. In brief, for blank nanosystems, 8 µL of DiR (20 mg/mL in ethanol) were added to the organic phase containing Vit E, SM and DOTAP (1:0.1:0.01 and 1:0.1:0.1, VitE:SM:DOTAP *w/w*), which was added to the aqueous phase as previously mentioned in Section 2.2. On the other hand, for pc (TP53TG1)-DSNs10% (10 µg plasmid) were labeled with NBD-SM. For that, initially DSNs10% were prepared by mixing Vit E, SM, 10 µg of NBD-SM (maintaining constant the final amount of SM) and DOTAP (1:0.1:0.1, VitE:SM/NBD-SM:DOTAP *w/w*) in ethanol and then add it to the aqueous phase (see Section 2.2). The plasmid pc (TP53TG1) was subsequently associated with NBD-SM labeled DSNs10% as described in Section 2.3.

HCT-116 cells were seeded in a glass coverslip in a 24-well plate at 1 × 10^5^ cells per 500 µL and after 24 h in culture, they were incubated with 0.1 mg of nanosystems (36 ng DiR or 40 ng of NBD dye per well). After 4 h of incubation at 37 °C, cells were washed with PBS, fixed with 4% paraformaldehyde, and the nucleus was stained with DAPI. The coverslips containing the cells were mounted and kept overnight at RT and then observed under confocal microscope (TCS SP5, Leica Microsystems GmbH, Heidelberg, Germany).

Flow cytometry analysis by fluorescence-activated cell sorting (FACS) was performed in order to further confirm NBD-labeled pc (TP53TG1)-DSNs10% (10 µg plasmid) internalization by HCT-116 cells. For that, HCT-116 cells were seeded at 1.5 × 10^5^ cells/well in 24-well plates and the next day were treated with NBD-labeled pc(TP53TG1)-DSNs (40 ng of NBD dye per well) for 4 h. Then cells were fixed with 0.4% paraformaldehyde solution and analyzed by FACS Calibur Flow Cytometer (BD Biosciences, San Jose, CA, USA) and results were analyzed using FlowJo software.

### 2.6. HCT-116 Cells Transfection

For fluorescence microscopy studies, 8 × 10^5^ HCT-116 cells were seeded in 600 µL of supplemented DMEM medium in 24-well plates. The next day, the medium was removed and the cells were washed with PBS and 300 µL of non-supplemented DMEM medium was added. Cells were transient transfected with 20 µL of pcDSNs associating 10 µg of pc(mCherry) for 4 h at 37 °C and 5% CO_2_. Then, the medium was removed and cells were washed with PBS and supplemented DMEM media was added. Twenty-four hours after, the cells were observed under fluorescence microscope (Leica M205 FCA).

### 2.7. Functional Assays

HCT-116 cells were seeded in Petri dishes (100 mm) at 3 × 10^6^ in 6 mL of complete DMEM medium one day before transfection. The day after, the medium was replaced with 5 mL of non-supplemented DMEM and cells were transient transfected with 200 µL of pcDSNs10% associating 10 µg of pcDNA empty vector (pc(empty)), pcDNA encoding wild type TP53TG1 (pc(TP53TG1)) or pcDNA encoding mutated TP53TG1 (pc(mutTP53TG1)). Four hours after transfection, the medium was removed, cells were washed with PBS and detached with trypsin to be used for the following assays:

Cell proliferation: 500 transfected cells were plated into each well in a 96-well plate and incubated at 37 °C and 5% CO_2_. At different time points (0, 24, 48, and 72 h) an MTT solution (5 mg/mL) was added, and after 3 h at 37 °C and 5% CO_2_ the formazan crystals were dissolved in 100 µL of DMSO. The absorbance was measured at 570 nm using an automatized micro plate reader (Multiscan Ex, Thermo-labsystems, Helsinki, Finland)).

Colony-forming: 100 pre-treated cells were seeded in 2 mL of supplemented DMEM medium in 12-well plates and 11 days later, an MTT solution (5 mg/mL) was added followed by 3 h of incubation at 37 °C. Then, the media was removed and the cells were kept at RT overnight. Afterwards, the cell plates were photographed and the colonies were counted using Open-CFU software.

Wound-healing: 1 × 10^6^ HCT-116 transfected cells per were seeded in 24-well plates in 500 µL of supplemented DMEM and after reaching complete confluence a wound was created through the monolayer using 10–100 µL pipette tips [35]. The wounds were monitored and photographed throughout the time course (0, 24, 48, 72, and 96 h) under a microscope (Leica, MS5). The wound area was measured using Image J software and migration rate was calculated normalized to the wound size at time 0 h (experiment start point).

### 2.8. Quantitative Reverse Transcription–Polymerase Chain Reaction (qRT-PCR)

HCT-116 cells were transfected with DSNs10%, without plasmid (blank) and associating 10 µg of pc(empty), pc(TP53TG1) or pc(mutTP53TG1), as previously described in Section 2.7. After 72 or 96 h of transfection, cells were collected and total RNA was extracted using Total RNA extraction kit (GeneJet RNA purification Kit, Thermo Scientific, Waltham, MA, USA) and cDNA was synthesized subsequently using 1 µg of RNA (High-Capacity cDNA Reverse Transcription Kit, Applied Biosystems, Foster City, USA). RNA expression was determined by quantitative RT-PCR (qRT-PCR) was performed using specific primers (Sigma, USA), described in Table 1, using PerfeCTa SYBR Green SuperMix Reagent (Quantabio, Beverly, MA USA) according to manufacturer’s protocol using AriaMx RealTime PCR system (Agilent Technologies, Cheshire, UK). GAPDH was used for the normalization as a house-keeping gene. Relative fold change was calculated as means of 2^−ΔΔt^ of expression.

### 2.9. Statistical Analysis

Statistical analysis was performed using SPSS, and graphs were prepared using GraphPad Prism. Statistical significance was determined by performing ANOVA followed by appropriate post hoc testing for multiple comparisons and Student’s *t*-test or Mann–Whitney U test for two-group comparisons. *p* values below 0.05 were considered statistically significant. All values are shown as average ± SEM.

## 3. Results

### 3.1. Preparation and Characterization of DSNs

In the present study, we propose the use of cationic lipidic nanosystems for the association and delivery of a plasmid expressing a tumor suppressor lncRNA, as a way to interfere with tumor growth and dissemination of colorectal cancer. To the best of our knowledge, only a few studies have attempted a similar strategy, by either expressing or silencing specific lncRNAs involved in tumorigenesis of such as prostate cancer [36] and metastasis-associated lung adenocarcinoma [37], respectively.

We report here the preparation of sphingomyelin nanosystems comprising DOTAP (DSNs) to provide a cationic charge able to mediate an optimal interaction with the negatively charged pcDNA. Previous studies have demonstrated the need for nanosystems with a small particle size (<150 nm) and a moderate positive surface charge (+10 to +50 mV) to potentiate oligonucleotides’ association by electrostatic interactions and intracellular delivery [38,39]. DSNs were prepared in one step, after mixing an ethanolic solution containing the lipids (Vit E, SM, and DOTAP) with ultrapure water, using a simple and fast homogenizer. An increasing percentage of DOTAP was added to the SNs (1% to 20%) to understand the effect of the cationic lipid concentration on the physicochemical properties of the nanosystems.

As reported in Table 2, control SNs had a mean particle size of 208 ± 2 nm, a polydispersity index (PdI) of 0.15, and a slightly negative surface charge (−10 ± 0 mV). After the addition of the cationic lipid DOTAP, a slight decrease of the particle size in all cases was observed, except for the higher concentration of 20%, probably due to an excess of DOTAP since the PdI was also higher for this composition. Despite that DSNs formulated with 1% and 10% DOTAP presented similar sizes and PdI, the last ones showed a more positive zeta potential, as expected. These DSNs were selected to study pcDNA association efficiency.

**Figure 1:**
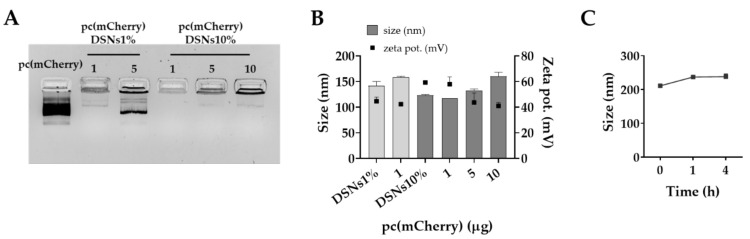
Association of pc(mCherry) to DSNs. (**A**) Association of pc(mCherry) (1 to 10 µg) to DSNs, prepared with 1% or 10% of DOTAP (DSNs1% and DSNs10%), determined by agarose gel electrophoresis. (**B**) Physicochemical properties of DSNs1% associating 1 µg of pc(mCherry) and DSNs10% associating 1, 5 and 10 µg of pc(mCherry). (**C**) Colloidal stability of DSNs10% associating 10 µg of pc(mCherry) in 1% FBS-supplemented DMEM, up to 4 h. (nm, nanometers; PdI, polydispersity index; zeta pot., zeta potential; mV millivolts).

Increasing amounts of pc(mCherry) (1 to 10 µg), used as model plasmid, were added to DSNs with 1% and 10% DOTAP by exhaustively mixing in 96-well plates. To understand if pc(mCherry) was efficiently associated with DSNs, samples were first analyzed by agarose gel electrophoresis and the migration of the unbound fraction was observed. DSNs with a higher amount of DOTAP (10%), in agreement with previous results in literature [26,40], were able to associate a higher amount of pc(mCherry) (Figure 1A). While most of the pc(mCherry) (5 µg) was in its free form for the formulation prepared with 1% of DOTAP, DSNs10% were able to associate at least 10 µg of pc(mCherry). The association of the model plasmid at 1 µg to DSNs1% did not lead to great alterations of the nanosystems’ physicochemical properties (Figure 1B). On the other hand, the associating of increasing amounts of plasmid (1, 5 and 10 µg) led to the increase of particle size, maintaining a low polydispersity (PdI < 0.2), and the decrease of DSNs surface charge, indicating that the plasmid was being successfully associated to DSNs surface, as it was confirmed by gel retardation assay. In addition, we observed that the pc(mCherry)-DSNs10% were stable in supplemented DMEM medium for at least 4 h at 37 °C (Figure 1C).

### 3.2. Nanosystems Cytotoxic Profile, Internalization Capacity and Transfection Efficiency

Despite improving the interaction of nanosystems with cells membrane, cationic compounds can also be harmful leading to toxic effects. Therefore, after characterizing the physicochemical properties of the nanosystems, the next move was to evaluate the toxicity profile of DSNs in HCT-116 cells. For that, the blank SNs and DSNs (1 and 10% DOTAP) as well as pc(mCherry)-DSNs10% (associating 10 µg of plasmid) were incubated with the colorectal cancer cells for 24 h at increasing concentrations (up to 5 mg/mL). As expected, DSNs containing a higher content of DOTAP (10%) presented a higher toxicity compared to the other blank nanosystems. Still, the plasmid (pc(mCherry)) adsorbed on the surface of DSNs10% showed a lower toxicity compared to the blank ones, which could be mainly due to shielding of DOTAP positive charges, which was previously hypothesized by the decrease of these nanosystems surface charge to neutral values.

**Figure 2:**
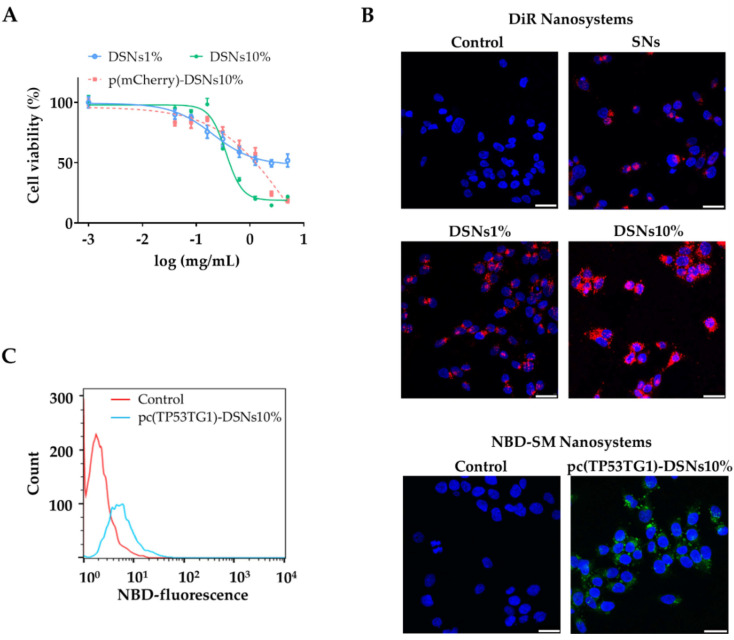
Nanosystems cytotoxic profile and internalization capacity on HCT-116 cells. (**A**) Blank nanosystems (SNs, DSNs1%, DSNs10%) and DSNs10% associating 10 µg of the model plasmid encoding mCherry (pc(mCherry)-DSNs10%) were incubated for 24 h with HCT-116 cells up to 5 mg/mL and cell cytotoxicity was determined by MTT. (**B**) Confocal microscopy images of internalized blank nanosystems labeled with DiR (red channel) and pc(TP53TG1)-DSNs10% (associating 10 µg plasmid) labeled with NBD-SM (green channel). Cell nuclei were counterstained with DAPI (blue channel). Scale bars correspond to 25 µm. (**C**) FACS analysis of HCT-116 cells positive to pc(TP53TG1)-DSNs labeled with NBD-SM (blue line) upon 4 h of incubation at 37 °C. Ultra-pure water was used as control (red line).

In order to elucidate whether the nanosystems could efficiently be internalized by colorectal cancer cells and deliver their payload, cell internalization experiments were carried out through confocal microscopy and FACS (Figure 2B,C). For confocal analysis two different approaches: (i) DSNs10% loading DiR (near-infrared lipophilic dye) and (ii) pc(TP53TG1)-DSNs10% prepared with SM covalently linked with NBD. As shown in Figure 2B (left panel), DSNs10% were rapidly internalized and accumulated in the HCT-116 cells’ cytoplasm after 4 h incubation, which was expected considering the ability of cationic lipids to improve the interaction of nanocarriers with the negatively charged cell membrane [41]. On the other hand, DSNs1% and specially SNs showed a decreased capacity to be internalized by HCT-116 cells. The second approach allowed us to ensure that we are tracking the nanosystems (in this case associating pc(TP53TG1)) and not the free dye, thereby validating the first one. Unlike DSNs, the ones associating plasmid presented a more neutral surface charge, nonetheless, and irrespective of the disposition of the pcDNA on the nanoparticles’ surface, both nanosystems (DSNs with 1 or 10% DOTAP) could be efficiently internalized by the cancer cells. This observation was further supported by FACS analysis of HCT-116 cells positive for the NBD after treatment with pc(TP53TG1)-DSNs10% (Figure 2C).

### 3.3. Transfection Efficiency in HCT-116 Cells

**Figure 3:**
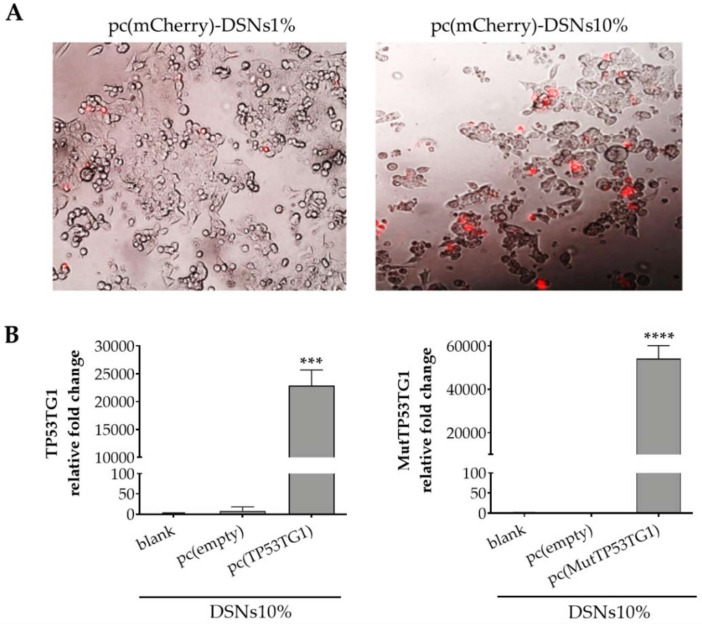
Transfection efficiency of DSNs in HCT116 cells. (**A**) Fluorescence microscopy images of mCherry (red signal) expression in cells transfected with a pc(mCherry)-DSNs1% and -DSNs10% for 24 h at 37 °C; (**B**) Expression of TP53TG1 and its mutated form (MutTP53TG1), in cells transfected with DSNs10% without plasmid (blank) or associating 10 µg of pc(empty), pc(TP53TG1), pc(MutTP53TG1), determined by qRT-PCR using specific primers. The relative expression of each gene was calculated as mean ± SEM of 2^-ddCT normalized to GAPDH. (*** *p* < 0.001; **** *p* < 0.0001).

To study the transfection efficiency of DSNs1% and DSNs10%, initially, a model plasmid encoding for mCherry was used since it allows a rapid observation under fluorescence microscopy. As can be observed in Figure 3A, after 24 h treatment the DSNs10% were more efficient in mediating the expression of the reporter fluorescent mCherry protein with respect to DSNs1%. This result is in agreement with the cell internalization assays (Figure 2B,C), as well as with other works in the field showing that there is a positive correlation between the higher content of the cationic component and the transfection efficiency [42,43].

In view of these results, we next investigated the expression of TP53TG1 lncRNA by qRT-PCR. Colorectal cancer cells were transfected with pcDSNs10% for 24 h. As control, the cells were transfected with blank DSNs10%, pc(empty)-DSNs10% or pc(MutTP53TG1)-DSNs10%. As shown in Figure 3B, transfection with pcDSNs10% associating the plasmid of interest (pcDNA4/TO expressing TP53TG1 or its mutated form) resulted in a dramatic increase in the relative fold change of TP53TG1 (*p* < 0.001) and of mutated TP53TG1 (*p* < 0.0001) expression, with respect to the control cells transfected with blank DSNs10% (without plasmid) or with the *p* (empty)-DSNs10%.

### 3.4. Tumor Suppressor Effects of pc(TP53TG1)-DSNs in HCT-116 Colorectal Cells

**Figure 4:**
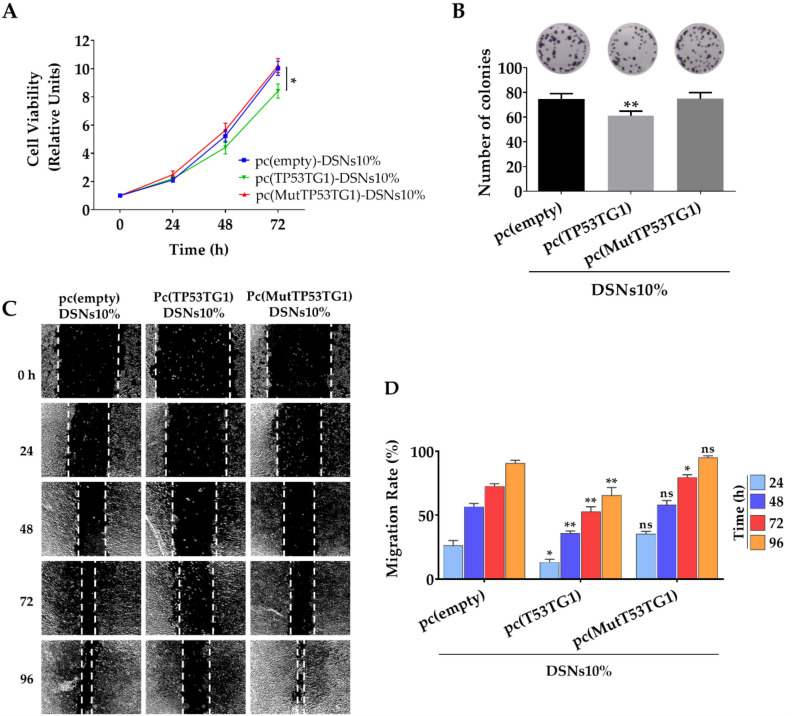
Tumor suppressor features of pc(empty)-DSNs10%, pc(TP53TG1)-DSNs10% and pc(MutTP53TG1)-DSNs10% in vitro. (**A**) HCT-116 cells’ viability determined each 24 h up to 72 h, after treatment with DSNs10%, associating different pcDNAs, for 4 h. Data is normalized to time point 0 h. (**B**) Effect of DSNs10%, associating different pcDNAs on the ability of HCT-116 cells to form colonies. HCT-116 cells were incubated with the nanosystems for 4 h and after 11 days the number of formed colonies was determined. (**C**) Wound healing assay was performed to evaluate the effect of the nanosystems on HCT-116 cell ability to migrate and close the wound. After creating a wound, the cells were treated with the nanosystems for 4 h (*n* = 3). The evolution of the wound was monitored up to 96 h through direct observation under light microscope and photos were taken. (**D**) The migration rate (wound healing assay) was calculated using the acquired photos and data was normalized against time point 0 h. The effects of pc(TP53TG1)-DSNs10% and pc(MutTP53TG1)-DSNs10% were compared to the ones produced by pc(empty)-DSNs10% at each time point. (ns, not significant; * *p* < 0.05, ** *p* < 0.01).

We previously showed that tumor suppressor activity of TP53TG1 is linked to its ability to block the tumorigenic activity of the RNA binding protein YBX1 [14]. In detail, we showed that TP53TG1 dampen HCT116 cell proliferation, invasiveness, and migration when it is stably expressed in these cells. Hence, herein we aimed to investigate if the delivery of TP53TG1 to HCT-116 cells using DSNs10% was able to induce the tumor suppressor features previously mentioned. As shown in Figure 4A, transient transfection with pc(TP53TG1)-DSNs10% resulted in a modest but significant decrease (*p* < 0.05) in cell viability, in relation to the values determined for control cells transfected with the empty or the mutated vectors. Next, we decided to evaluate the ability of HCT-116 cells to form colonies. As depicted in Figure 4B, transfection with pc(TP53TG1)-DSNs10% interfere with the colony-forming capacity of HCT-116 cells that had a significantly (*p* < 0.01) lower density of colonies than cells transfected with the empty or the TP53TG1 mutated form. Finally, a wound-healing assay was also performed, showing that cells transfected with DSNs10% associating the plasmid encoding the lncRNA TP53TG1 also had a significantly lower migration rate than the control and mutated TP53TG1, according to the final size of the wound 48 to 96 h after transfection (Figure 4C,D).

## 4. Discussion

NcRNAs are a large group of transcribed RNAs including miRNAs, Piwi-interacting RNAs, siRNAs, and lncRNAs [44,45]. The role of small ncRNAs in genome regulation and diseases is broadly studied [46]. Nonetheless, recent studies have concluded that the majority of RNAs transcribed by the human genome are in the form of long ncRNAs [47] and the number of these RNAs with validated functions is growing exponentially. Of note, lncRNAs are known to be implicated in nearly all diseases, including cancer, which is associated with the large quantity of lncRNAs with regulatory properties as chromatin modification, splicing, and transcriptional regulation [48]. LncRNAs normally function as protein scaffolds so they interact with DNA or RNA through nucleic-acid base pairing or with proteins through higher-order RNA structures [49]. However, lncRNAs may undergo promoter mutations or abnormal expression in tumors, and also go through epigenetic variations resulting in enhanced susceptibility to cancer [50]. Among tumor suppressor lncRNAs are CASC15-S [51], Uc.283+A [52], and BRAF-activated lncRNA. In addition, p53 related tumor suppressor lncRNAs such as MT1JP [53], LincRNA-p21 [9], and TP53TG1 [14] are shown to have differential expression in tumors. LncRNAs’ functions seem to be cell-type specific and mainly limited to only a single cell type [54]. Thus, tumor suppressor lncRNAs have potential roles in the diagnosis and treatment of specific tumors. In a previous study by Diaz-Lagares et al., in vitro and in vivo assays showed that TP53TG1 has tumor-suppressor activity in colorectal cancer, increasing the sensitivity to DNA-damaging agents used in colorectal cancer (e.g., 5-fluorouracil + oxaliplatin) and small targeted molecules, such as PI3K and AKT inhibitors [14]. Considering that colorectal cancer is the second most deadly type of cancer and that the number of new cases keeps rising [55], the need for new and more effective treatments is of extreme urgency. In this sense, we decided to further exploit the potential of lncRNA TP53TG1, unveiled by Diaz-Lagares et al. As it is well known, genetic material faces different obstacles upon administration to reach the target cell, such as the degradation by nucleases, instability in the systemic circulation and inability to enter cells. In this regard, different types of nanocarriers, either inorganic, polymeric or lipidic, have been developed to deliver a variety of polynucleotides aimed for the treatment of several human conditions [56,57]. There are already some examples showing that nanotechnology can successfully overcome such obstacles, such as Onpattro^®^ (Alnylam^®^ pharmaceuticals), Spikevax (Moderna Inc.), Vaxzevria (Astrazeneca/Oxford) which are approved by the U.S. Food and Drug Administration (FDA) and the European Medicines Agency (EMA). Therefore, in the present work, we aimed at developing a safe carrier for TP53TG1 delivery, in order to boost its action on modulating tumor behavior and reducing its progression. Among the developed vehicles, lipidic nanosystems, namely cationic liposomes are certainly the most cited in the literature for polynucleotide delivery [21]. Among the different cationic lipids, DOTAP is one of the most largely used in liposomes [58,59] but also lipid nanoparticles (LNPs) [60] and nanoemulsions [26,61,62] aimed for polynucleotides delivery. We have previously determined that DOTAP-miRNA lipoplexes, in a specific ratio, could be encapsulated in SNs leading to better results in terms of stability, internalization as well as transfection when compared to ST [28]. Therefore, herein we took advantage of DOTAP along with Vit E and SM to construct a lipidic nanosystem for the delivery of a plasmid vector, encoding lncRNA TP53TG1, to HCT-116 colorectal cancer cells. The presence of a net positive charge on the carrier’s surface, conferred by DOTAP, allows a spontaneous interaction with the negatively-charged plasmid [63]. Importantly, this cationic lipid benefits from a high complexation efficiency as well as the presence of a biodegradable chemical ester bond that assures its biodegradation [64] and, consequently, the possible toxic effects should be transient. In addition, it has been under evaluation in different clinical trials (NCT00059605, NCT01455389, NCT04580771, NCT04287868).

Initially, we studied the influence of increasing concentrations of DOTAP (1 to 20%) on SNs to find the optimal amount. The nanosystems were prepared by the simple and fast method of homogenization using an ultra-turrax upon the addition of the organic phase into the aqueous phase. As shown in Table 2, the nanosystems comprising DOTAP (DSNs) up to 10% presented a smaller size (< 150 nm) than SNs (208 nm) while maintaining a low PdI (< 0.2). In addition, the increase of the zeta potential from negative to positive also indicated that DOTAP was efficiently incorporated in SNs. A similar effect was observed by Martini et al. [65]. However, DOTAP at 20% produced bigger particles (230 nm) and less homogeneous (polydispersity index, PdI 0.3) probably due to an excess of this lipid regarding the rest of the nanosystem composition. Thus, DSNs1% and DSNs10% were selected to study pcDNA association efficiency. As determined by gel retardation assay, the nanosystems containing the highest percentage of DOTAP (DSNs10%) successfully associated more pcDNA. In addition, pcDNA (10 µg) association resulted in an increase of particles’ size due to the presence of adsorbed plasmids (≈ 7000 bp) on particle’s surface. Still, it did not compromise the homogeneity of the formulation (PdI < 0.2). In a similar context but using a thin-film hydration method, Duan et al. prepared cationic liposomes composed of vitamin E, cholesterol, and DOTAP for siRNA delivery against hepatitis C virus [66]. DOTAP liposomes were able to successfully transfect functional siRNAs into hepatocytes both in vitro and in vivo with low injection dose requirements and low toxicity, demonstrating the potential of this approach.

Before performing any in vitro assays, we confirmed that the pc(mCherry)-DSNs10% were stable in supplemented cell culture medium, ensuring that the posterior studies would not be affected by physicochemical changes of the nanosystem physicochemical properties. Naturally, the following step was to study the cytotoxic profile of the nanosystems in order to guarantee that DSNs concentrations selected for the functional assays would not lead to toxicity. As stated in other studies using DOTAP for nucleic acids’ delivery [28,66,67,68], we further showed that the DSNs designed in this work, namely DSNs10%, could be easily internalized by HCT-116 cells in vitro. On the contrary, neutrally-charged SNs (without DOTAP) were weakly internalized by the cells. The interaction of cationic nanocarriers with cells is mediated by electrostatic interactions between the positively-charged surface of the NPs and the negatively charged cells membrane. The higher capacity of DSNs10% to be internalized in comparison to DSNs1% was further demonstrated by the transfection assay performed with DSNs associating a plasmid encoding fluorescent mCherry, which showed a higher number of fluorescent cells after incubation with DSNs10%. Considering the collected information, this formulation was selected for further studies. The efficient delivery and release of the plasmids once inside the HCT-116 cells was demonstrated by the high levels of TP53TG1 and mutated TP53TG1 gene expression as determined by qRT-PCR, which also demonstrate the internalization of pcDSNs10%.

The main aim of this work was to deliver the tumor suppressor features of TP53TG1 lncRNA to cancer cells. The pc(TP53TG1)-DSNs10% transfection to HCT-116 cells was successful to do so by showing a significant decrease of the proliferation and migration rates of this cell line, in comparison to the treatment with DSNs10% associating an empty vector as well as a vector expressing the mutated form of TP53TG1 lncRNA. The same tendency was observed in HCT-116 cells colony-forming capacity after pc(TP53TG1)-DSNs10% treatment. The mutated form presented a point mutation by site-directed mutagenesis at the second A and fourth C of all the CACC motifs (CACC>CTCG) present in the TP53TG1 wild-type sequence, which are responsible for the high-affinity YBX1 binding. Therefore, the results corroborate that losing the TP53TG1 lncRNA binding site to YBX1 leads to the impairment of TP53TG1 anti-proliferative capacity. These results were consistent with Díaz-Lagares et al. findings where the tumor suppressor effects of TP53TG1 lncRNA and its associated mechanisms to exert anti-tumor properties were first demonstrated [14]. Despite that the anti-proliferative capacity of pc(TP53TG1)-DSNs10% might seem lower when compared to that observed in a previous work by Díaz-Lagares et al., a direct comparison to that study is not feasible since it was performed using lipofectamine as a transfection agent. Although being widely used for transfection and comparative purposes, lipofectamine is only approved for research and not for clinical use. On the other hand, it is important to take into consideration that in our study we have used HCT-116 cells transiently transfected with the plasmid encoding for TP53TG1, while Díaz-Lagares et al. in their previous study used the HCT-116 cell line stable transfected with a continuous expression of TP53TG1, a methodology that allows the visualization of clearer effects than transient transfections. Nonetheless, the results shown in this work provide evidence that our nanosystems composition, namely vitamin E and sphingomyelin and only a small percentage of cationic lipid can be used as safe vehicles for the delivery of oligo-nucleic acid therapeutics to cells. In order to improve the efficacy of our nanosystems, in the future, other cationic lipids such as ionizable or zwitterionic lipids have been recently used in different cationic nanocarriers for polynucleotide delivery [60,69] as well as helper lipids could be included in our nanosystem composition. Another option to take into consideration is to modulate our nanosystems to actively target colorectal cancer cells so that they can improve their accumulation at the tumor site. For that we could include in our nanosystems composition a PEGylated lipid conjugated to a peptide as uroguanylin (ligand of guanylyl cyclase receptor, expressed in metastatic colorectal cancer cells) which our group has recently demonstrated to be fast and efficiently internalized in vitro as well as to improve the therapeutic efficacy of the encapsulated chemotherapeutic drug by significantly decreasing the tumor volume when administered in vivo [31]. On the other hand, the transient effects of delivered TP53TG1 lncRNA could be overcome by treatment with higher doses and even multiple administrations, yet, the latter should be further examined in vitro with higher doses and perhaps longer treatment periods as well as in in vivo settings.

## 5. Conclusions

To our best knowledge, this is the first study reporting the therapeutic value of TP53TG1 lncRNA in colorectal carcinoma in combination with nanotechnology. Cationic sphingomyelin nanocarriers were tailored to deliver a plasmid encoding TP53TG1 lncRNA to human colorectal cancer cells. The carriers were optimized in terms of DOTAP amount for an efficient transfection. DSNs containing 10% DOTAP presented a high capacity to associate the plasmid as well as to lead to an efficient transfection demonstrated by the levels of TP53TG1 gene expression. In addition, pc(TP53TG1)-DSNs10% significantly decreased the HTC-116 cells proliferation and migration rates in comparison to control treatments, as well as the capacity of these cells to form colonies. It is noteworthy that the results obtained in this study follow the same trend as previous results showing the tumor suppressor effects of lncRNA TP53TG1. We envision that this delivery system can be further modulated to efficiently deliver a range of different oligonucleotides to other cancer cell types with further modification. Further studies are needed to understand the required dose for a greater and sustained effect both in vitro and in vivo.

## Figures and Tables

**Table 1:** List of primers used for qRT-PCR.

Assay	Forward	Reverse
wild type TP53TG1	CTTTCCTTTAATCTTCGGAGGC	TGCCAGCTCTCAGAGTCCTT
mutated TP53TG1	CTCGAGGCGAACACTTACTC	TCTCAGAGTCCTTCGAGGTT
GAPDH	TCTTCCAGGAGCGAGATC	CAGAGATGATGACCCTTTTG

**Table 2:** Characterization of SNs and DSNs with increasing percentages of DOTAP.

Composition (DOTAP %)	Size (nm)	PdI	Zeta Pot. (mV)
SNs	208 ± 2	0.15	−10 ± 0
DSNs1%	136 ± 1	0.16	+26 ± 1
DSNs5%	141 ± 2	0.16	+51 ± 4
DSNs10%	142 ± 2	0.17	+47 ± 2
DSNs20%	229 ± 2	0.27	+48 ± 3

SNs: Sphingomyelin nanosystems; DSNs: DOTAP-sphingomyelin nanosystems; nm: nanometers; PdI: polydispersity index; zeta pot., zeta potential; mV millivolts.

## Data Availability

The data presented in this study are available on request from the corresponding author.
